# Reproductive chemical database: a curated database of chemicals that modulate protein targets regulating important reproductive biological processes

**DOI:** 10.1186/s13578-024-01261-1

**Published:** 2024-06-06

**Authors:** Yuedi Cao, Geng G. Tian, Xiaokun Hong, Qing Lu, Ting Wei, Hai-Feng Chen, Ji Wu

**Affiliations:** 1 Key Laboratory for the Genetics of Development & Neuropsychiatric Disorders (Ministry of Education), Bio-X Institutes, Shanghai Jiao Tong University, Shanghai, 200240 China; 2 School of Agriculture and Biology, Shanghai Jiao Tong University, Shanghai, 200240 China; 3 State Key Laboratory of Microbial Metabolism and Joint International Research Laboratory of Metabolic & Developmental Sciences, National Experimental Teaching Center for Life Sciences and Biotechnology, School of Life Sciences and Biotechnology, Shanghai Jiao Tong University, Shanghai, 200240 China; 4 Key Laboratory of Fertility Preservation and Maintenance of Ministry of Education, Ningxia Medical University, Yinchuan, 750004 China

**Keywords:** Database, Chemical, Reproduction, Function prediction, Target protein screening

## Abstract

Recent studies have shifted the spotlight from adult disease to gametogenesis and embryo developmental events, and these are greatly affected by various environmental chemicals, such as drugs, metabolites, pollutants, and others. Growing research has highlighted the critical importance of identifying and understanding the roles of chemicals in reproductive biology. However, the functions and mechanisms of chemicals in reproductive processes remain incomplete. We developed a comprehensive database called the Reproductive Chemical Database (RCDB) (https://yu.life.sjtu.edu.cn/ChenLab/RCDB) to facilitate research on chemicals in reproductive biology. This resource is founded on rigorous manual literature extraction and precise protein target prediction methodologies. This database focuses on the delineation of chemicals associated with phenotypes, diseases, or endpoints intricately associated with four important reproductive processes: female and male gamete generation, fertilization, and embryo development in human and mouse. The RCDB encompasses 93 sub-GO processes, and it revealed 1447 intricate chemical–biological process interactions. To date, the RCDB has meticulously cataloged and annotated 830 distinct chemicals, while also predicting 614 target proteins from a selection of 3800 potential candidates. Additionally, the RCDB offers an online predictive tool that empowers researchers to ascertain whether specific chemicals play discernible functional roles in these reproductive processes. The RCDB is an exhaustive, cross-platform, manually curated database, which provides a user-friendly interface to search, browse, and use reproductive processes modulators and their comprehensive related information. The RCDB will help researchers to understand the whole reproductive process and related diseases and it has the potential to promote reproduction research in the pharmacological and pathophysiological areas.

**Graphical Abstract:**

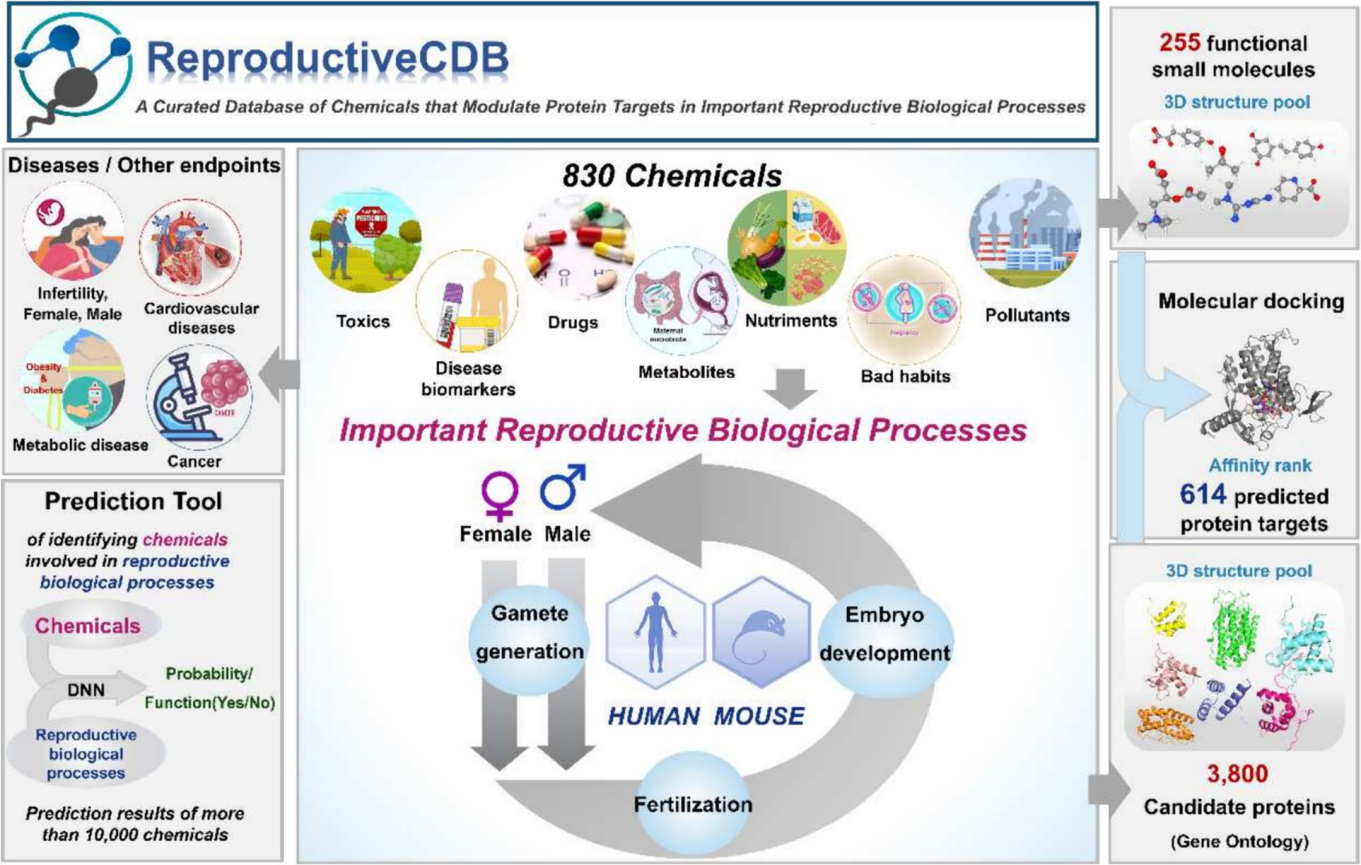

## Background

In mammals, the reproductive process begins with the selection of the fittest gametes, which then follow a unique developmental pathway, participate in fertilization, and ultimately give rise to a viable embryo capable of developing into a new life [1]. These complex and highly organized activities require a range of factors deliberately selected to meet the dynamic needs of life [2–4], and disruptions by chemicals may cause severe outcomes, such as abnormal development [5–7], infertility [8], and even transgenerational or multigenerational effects [2, 9, 10]. The use of preconception antidiabetic drugs in men, such as metformin, is associated with birth defects in their offspring [10, 11]. Decades of research have highlighted the critical role of metabolism in preimplantation development [12–14]. In a recent study, Zhao et al. observed differences in 76 metabolites between two-cell embryos and blastocysts during mouse embryo development using ultra-sensitive metabolomics. L-2-hydroxyglutarate affects embryo development by inhibiting the erasure of H3K4me3 methylation, which is involved in metabolic pathways [15]. Besides direct chemical exposure or transformation, the maternal microbiome can also modulate fetal neurodevelopment in mice, probably through signaling by microbially modulated metabolites to neurons in the developing brain [16, 17]. Organisms regulate the functions of large macromolecules, such as DNA and protein, to cope with the constant changing environment, through interactions occurring between macromolecules and small molecules (i.e., small organic molecules). Small molecules are responsible for the essential processes of life, such as energy production, signaling, and pathogen eradication. Therapeutic drugs are often small molecules, which regulate the functions of macromolecules by interacting with them. Therefore, understanding whether a small molecule can interact with a macromolecule at the cellular and molecular levels, especially in the fields of medicine and molecular biology, is important [18].

Chemicals have been extensively used in many aspects on the basis of their effect on reproductive processes mentioned above. The detection of the metabolomics during important reproductive processes will help in further understanding the metabolic regulation of development [15]. Additionally, the microenvironment could contain metabolomics biomarkers for some diseases, providing new knowledge to improve the efficiency of critical techniques such as in assisted reproductive technology or to predict and improve outcomes in reproductive medicine [19]. In the field of drug development for the reproductive system, a research team developed an optimized TDI-10229 inhibitor for soluble adenylyl cyclase, which is crucial for mouse spermatogenesis [20]. This breakthrough demonstrates the possibility of two groundbreaking human contraception methods: non-hormonal male contraception and on-demand pharmacological contraception. Drug-induced or chemical-induced reproductive toxicity can lead to extremely serious outcomes, and reproductive toxicity accounts for 3% of cases of drug withdrawal/discontinuation [21]. Many studies used machine learning methods to identify and characterize compounds with reproductive toxicity from chemicals [22–24].

There are some large chemically-indexed databases, such as PubChem [25], Drugbank [26], ChEMBL [27], KEGG [28], and Comparative Toxicogenomics Database (CTD) [29], which do not focus on reproduction. Therefore, these databases have disadvantages, including incomplete information and difficulty in obtaining target information on reproduction. However, some databases include small molecules of reproductive toxicity, such as Toxin and Toxin-Target Database (T3DB) [30], TOXRIC [31], European Chemicals AgencyC&L Inventory, and the OECD eChemPortal. These data provide a single endpoint, such as positive (reproductive toxicants) or negative (non-reproductive toxicants), instead of the molecular mechanism/phenotype and do not reflect the diversity of phenotypes. Therefore, the application of these data for predicting toxicity is limited. As an integral part of biological processes in living organisms and the foundation of target identification and drug discovery, chemical–protein interactions are determined by computational methods. These methods circumvent the time and cost associated with experimental approaches. Information on these interactions is dispersed over many databases, texts, and prediction methods. However, researchers seeking a comprehensive understanding of important reproductive biological processes face a challenge in gathering relevant information because it is scattered across thousands of publications. Moreover, there is currently no database available that consolidates chemicals from various data sources that are pertinent to these biological processes.

To overcome these limitations, we developed the Reproductive Chemical Database (RCDB) (https://yu.life.sjtu.edu.cn/ChenLab/RCDB) of chemicals and their predicted protein targets that modulate corresponding processes. The RCDB has three key features. (i) This database is a comprehensive and integrative platform for reproductive chemicals. The RCDB is a versatile database that provides a multitude of annotations surrounding small molecules, including their basic information, phenotypes, related diseases, and predicted protein targets. The Gene Ontology (GO) biological process is used to define and standardize the functional periods of these phenotypes and diseases, with annotations that are restricted to the important reproductive processes. The RCDB is an extensive collection of annotations derived from over 600 literature sources, and it comprises more than 800 chemicals. These annotations provide a comprehensive view of their roles in the reproductive process and related diseases as reported in the literature. Additionally, this database offers excellent candidate molecules, such as metabolomics data that significantly change during the preferred reproductive biological process and markers for some diseases, and even new chemicals lacking functional evidence. (ii) The RCDB uses molecular docking to efficiently narrow the screening scope of target proteins. This database allows creative use of reverse docking to identify suitable protein targets for small molecules according to their functions in the GO biological processes. (iii) The RCDB provides datasets for researchers interested in investigating small molecules in important reproductive processes. The RCDB provides comprehensive, machine learning-ready sub-datasets for “how the endpoints occurred” instead of “endpoints that have already occurred”, which can be downloaded and used as input/output for machine learning models.

## Construction and content

### Data collection and content

**Fig. 1:**
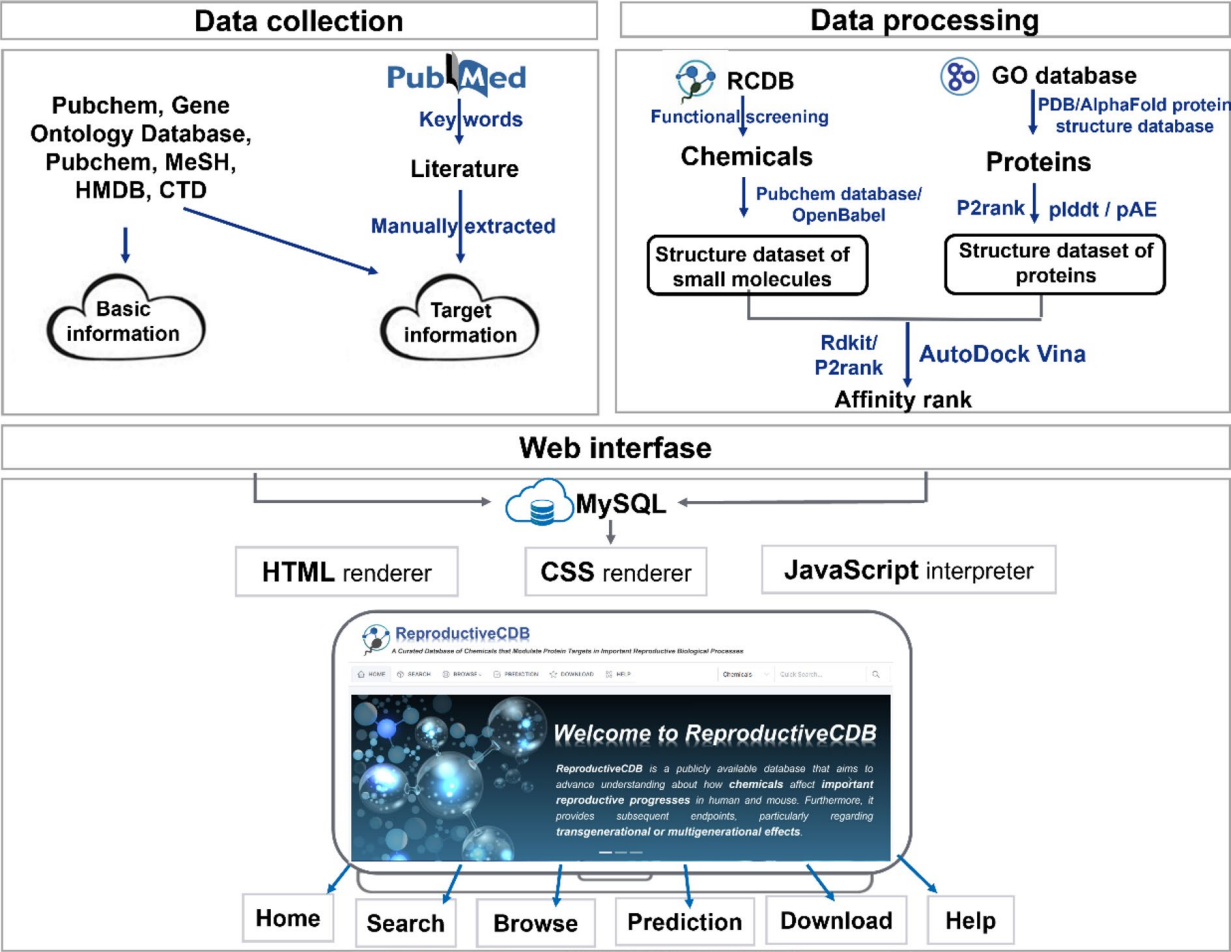
Overview of data collection, processing, and database interface. Data collection involves obtaining target information through using publicly available databases. Data processing primarily involves the molecular docking analysis of chemicals and proteins, which is achieved by using AutoDock Vina software. The web interface is mainly divided into six parts, comprising “Home,” “Search,” “Browse,” “Prediction,” “Download,” and “Help”

The general process of data collection is illustrated in Fig. 1. Data collection primarily falls into two major categories. The first category involves obtaining basic information and some target information by leveraging public databases, while the second involves the compilation of target information through a manual literature review. We searched PubMed using a list of keywords, such as ‘chemicals’, ‘compound(s)’, ‘metabolite(s)’ and ‘small molecule(s)’, and reproductive processes related keywords including but not limited to ‘embryo development’, ‘oogenesis’, ‘spermatogenesis’, ‘fertilization’ and their synonyms, as well as species information (‘human’ OR ‘mouse’ OR ‘mice’) and finally obtained more than 1000 related publications. To reliably collect the information, professional biocurators who major in reproductive biology engage in the manual curation of scientific literature. We employ a system of peer review and regular discussions to guarantee the data's accuracy and careful to focus on collecting chemical-induced phenotypes to keep our resources focused on chemical-centric data. In terms of data collection, we conduct a professional manual curation of the acquired literature, aiming to extract target information that primarily includes chemical molecules, species, associated biological processes, relevant diseases and information, summaries derived from the original texts, species, and PMID. Furthermore, we employ standardized definitions of the relationships between small molecules and biological processes by utilizing terms such as ‘increase’, ‘decrease’, ‘affect’ and ‘not’. Furthermore, to define phenotypes, we incorporated the GO database as a vocabulary source for non-disease biological outcomes. The GO resource is a widely recognized, community-accepted vocabulary with accession identifiers, ensuring that chemical–phenotype interactions are computable and interoperable with other databases. With regard to disease annotation, various diseases are standardized using the standard terminology in Medical Subject Headings. We referred to common methods of definition for the definition of generation [32]. Effects in the F1 and F2 generations are considered multigenerational, and effects in the F3 generation are considered transgenerational.

### Data processing

#### Data generation of potential targets in humans and mice

Two lists of genes of important biological processes in humans and mice were created from the GO database [33]. Genes without a UniProtKB ID were discarded because the AlphaFold Protein Structure Database only contains models with an entry in UniProtKB [34]. All protein models in PDB format were downloaded from the Protein Data Bank database. If not available, the models will be downloaded from the AlphaFold Protein Structure Database downloads section on 12 October 2022. Selected genes without a model were a consequence of their products’ length being larger than 2700 residues, and these were also discarded because AlphaFold models were not available for such lengths. Binding pocket prediction was performed using P2Rank [35] with standard settings. Pockets with a probability score (as provided by P2Rank) > 0.1 were considered as candidates for binding sites. In each model, the pocket with the highest probability score was selected as the binding site. Structures without predicted pockets or predicted pockets with a probability score < 0.1 were discarded. To further assess and shelter the AlphaFold source model quality, the predicted Local Distance Difference Test (pLDDT) [36] score of each α-carbon was extracted from the Protein Data Bank files. The proportion of residues with a pLDDT score > 70 (described as the threshold for good backbone prediction) [37] was then calculated. Only models with at least half of its total residues with a pLDDT score > 70 were considered for docking. Additionally, to assess the local quality of the binding pocket, the residues predicted to be part of the pocket by P2Rank were considered. Only models in which at least half of the residues in the pocket had a pLDDT score > 90 were kept. This stricter threshold was chosen because residues with a pLDDT score > 90 can be interpreted as having high quality and correct side-chain orientation [37]. Finally, the predicted aligned error (pAE) of the pocket residues was also analyzed. Therefore, the mean pAE of each residue of the pocket (as specified by P2Rank) with the rest of the residues of the pocket was calculated, and the overall mean pAE was obtained. Any model with a mean pocket pAE > 5 Å was discarded.

#### Generation of human and mouse datasets of functional ligands

To choose ligands, we conducted a meticulous search within the RCDB, and specifically focused on compounds associated with reproductive biological processes. Ligand structures were retrieved from PubChem [25] as three-dimensional (3D) SDF files whenever available. If 3D files were not accessible, we downloaded them as 2D SDF files and used Open Babel to generate the definitive 3D conformations [38]. The size of the binding box for each ligand was optimized according to Feinstein and Brylinski [39], using a radius of gyration to box side ratio of 0.35, and rounding up to the nearest integer. The radius of gyration for each ligand was calculated using the Python RDKit library Descriptors3D module.

### Reverse docking for functional ligands

Docking simulations were performed using AutoDock Vina 1.1.2 [40]. The exhaustiveness parameter was set to 8 and the energy was set to 2. The search box center was chosen from the P2Rank predictions, and its size was calculated for each ligand as described above. Ten docking repetitions were performed with different random seeds for each receptor and ligand pair. The best mode for each pair was chosen from the lowest docking energy of all the repetitions. This resulted in a matrix of n ligands by m receptors, with the best possible energy for each pair. The results were visualized using Jsmol [41].

### Website implementation

The RCDB website was implemented by using Hypertext Markup Language, Cascading Style Sheets, and JavaScript running on top of a MySQL database.

### Identification of interactions between chemicals and important reproductive biological processes based on machine learning

**Fig. 2:**
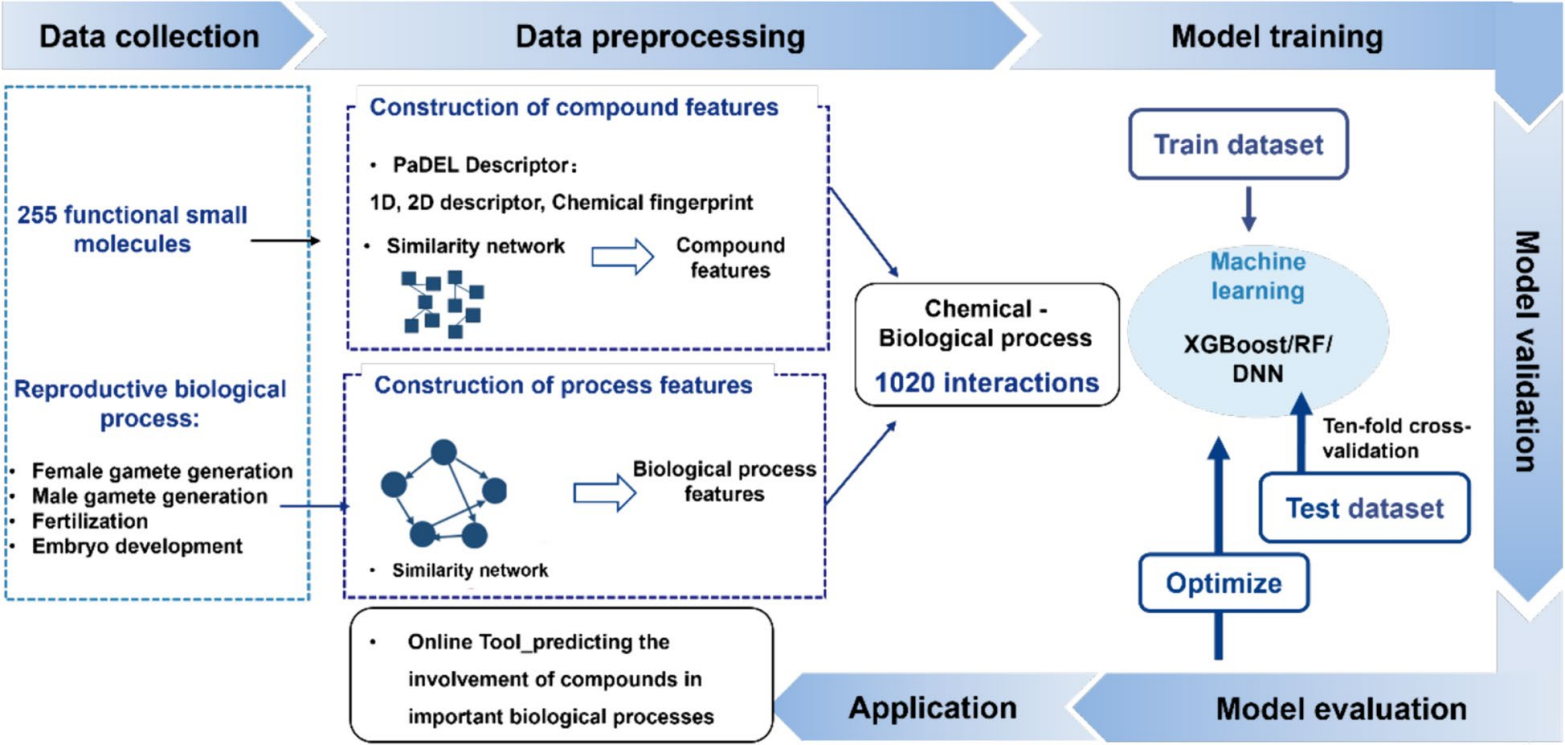
Workflow for constructing machine learning (ML) models

Chemicals and their interactions with important reproductive biological processes were extracted from the RCDB. The RCDB provides positive and negative chemical–biological process interactions (CBIs). Any combination of chemical and biological processes constitutes a CBI. An example of this combination is that CBI (1,1) is constructed by chemical 1 and biological process 1. The features of CBI (1,1) are the combination of the features of biological process 1 and compound 1, and then we apply a z-score for normalization. Finally, we obtained 1020 CBIs in the mouse from the RCDB. Among these CBIs, 320 CBIs were positive and the others were negative. To show the convenience of the RCDB, we developed a deep learning-based method to predict CBIs. The workflow of our method is shown in Fig. 2.

There were three steps in our method for data preprocessing: construction of chemical features, construction of biological process features, and CBIs. To construct chemical features, the 2D SDF files of the chemicals were input into PaDEL-Descriptor software [42] to obtain 1444 dimensional 1D and 2D descriptors and 12 types of fingerprints for each chemical. We then calculated various compound–compound similarities by the Jaccard index [43, 44]. To construct biological process features, we assessed the semantic similarity between GO terms, which was based on an encoding method that quantitatively represents the semantic content (biological meanings) of GO terms. The encoding method integrates the semantic contributions from ancestral terms, including the specific term itself, within the GO graph [45]. To implement tenfold cross-validation, we randomly divided the positive and negative samples into 10 groups. One of the positive groups and one of the negative groups were then used as the testing set, and all of the other groups were used as the training set. This process ensured that the ratio of positive and negative samples was the same between the training set and the testing set.

### Collection and treatment of mouse early embryos

All animal experiments followed procedures approved by the Institutional Animal Care and Use Committee of Shanghai Jiao Tong University. Six- to eight-week-old ICR/CD1 mice were collected and intraperitoneally injected with 10-IU pregnant mare serum gonadotropin (PMSG), followed by a human chorionic gonadotropin (hCG; 10 IU) injection 48 h later. Then, we mated those superovulated females with ICR/CD1 males and collected the zygotes from the oviducts of female mice at 20 h post-hCG injection and cultured in KSOM medium as control group. The treatment group of octyl octanoate dissolved in 0.1% ethanol were added to reach a final concentration of 0.5 μM in the culture medium. Early 2-cell and blastocyst stage embryos were harvested at 34 h, and 4 days post-hCG injection, respectively.

## Results

### Overview of the RCDB

**Fig. 3:**
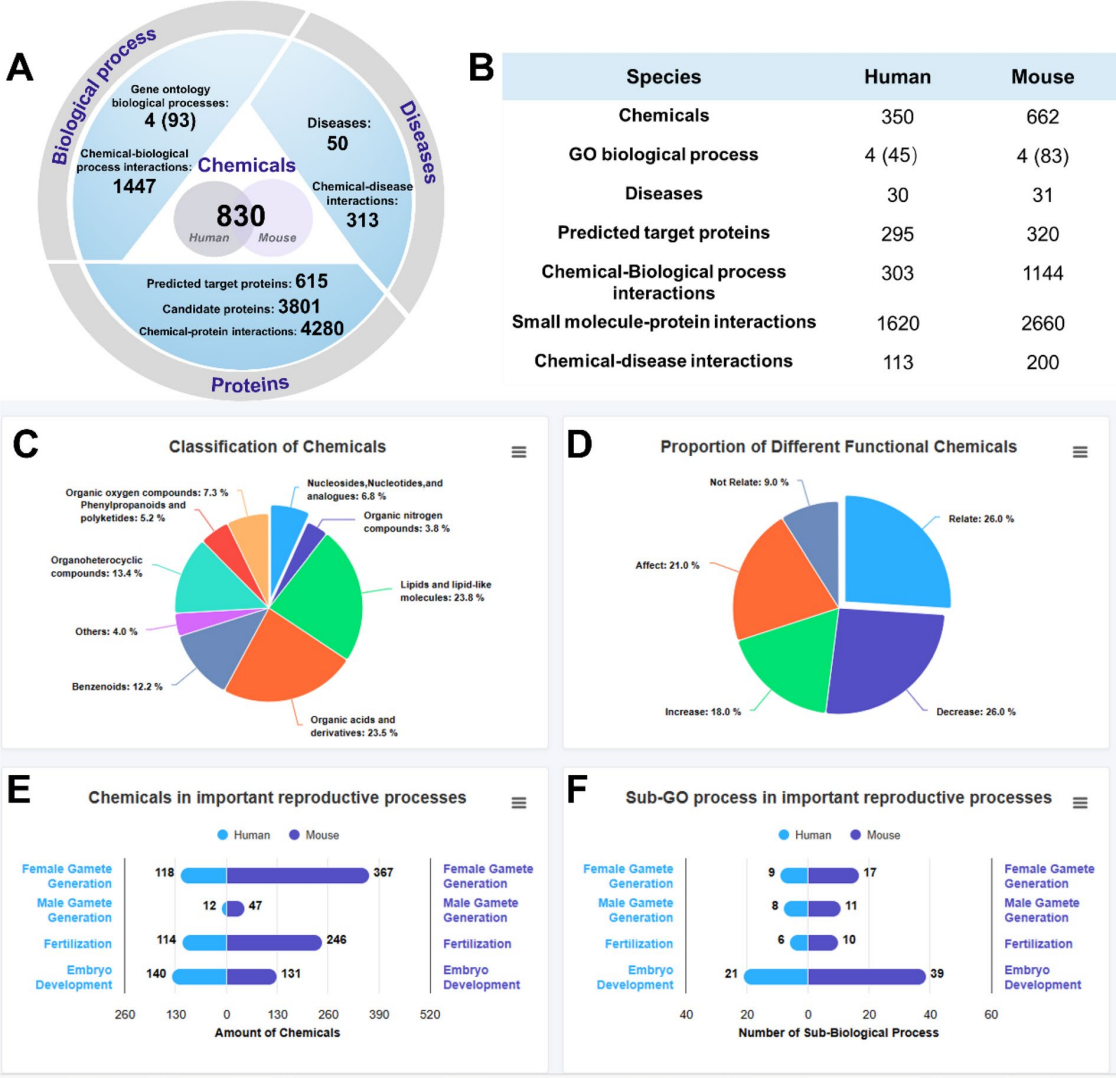
Statistical results of data stored in the RCDB. **A** Overview of the statistical situation of the database **B** Statistical results of humans and mice in the database. **C** Chemical classification in the database. **D** Proportion of different functional chemicals. **E** Number of chemicals in important reproductive processes. **F** Number of sub-GO processes of important reproductive processes

The RCDB contains a map of 350 and 662 related chemicals in humans and mice, respectively, involving 30 diseases and 45 sub-GO process in human, and 31 diseases and 83 sub-GO process in mouse (Fig. 3B). These markers were derived from 4124 annotation entries collected from more than 600 published reports. On the basis of the focus on chemical properties and functionality, we classified compounds using pie charts (Fig. 3C). These graphs showed that the chemicals collected in the database exhibited a rich diversity. This finding suggests that the occurrence and regulation of reproductive processes require the participation of multiple metabolites, and various compounds in the environment may have a considerable effect on reproductive processes. Additionally, the diverse definitions of functionality and the relatively balanced distribution can meet the different requirements of a wider range of users. The amount of chemicals in important reproductive biological processes and the sub-GO process in important reproductive processes are shown in bar plots (Fig. 3F). The associated compounds and sub-GO processes were more abundant than the other three processes owing to the complexity of embryo development.

### Web interface

**Fig. 4:**
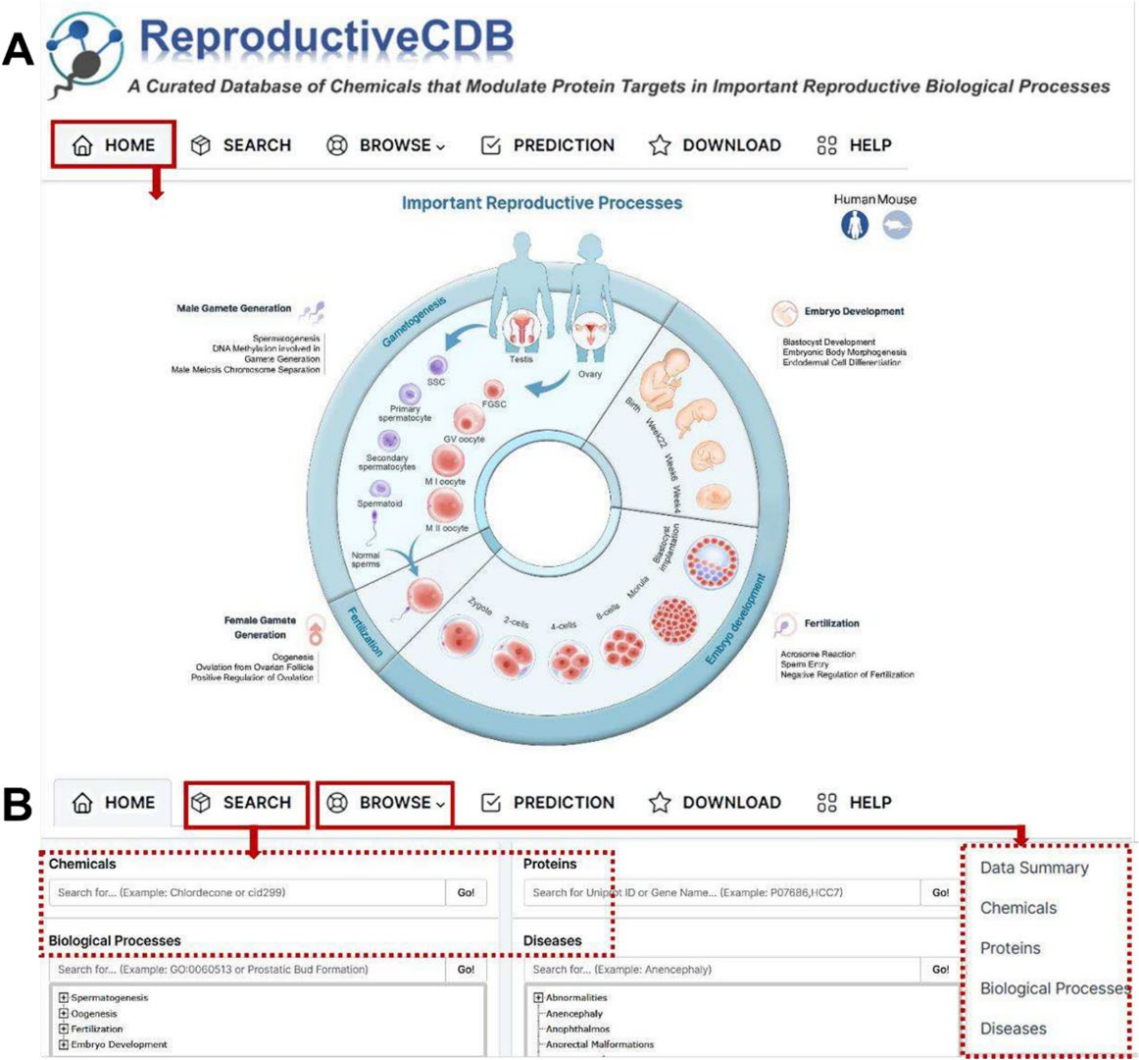
Schematic workflow of the RCDB. **A** The web images in the home page allow quickly search for important reproductive biological processes. **B** The “Browse” and “Search” pages allow the users to browse and search chemicals

The RCDB’s public user interface allows intuitive browsing and searching of any data in the database. On the “Home” page, users can quickly explore reproductive processes by clicking on hyperlinks embedded in the web images—Important reproductive processes in the human or mouse (Fig. 4A). After clicking on the icons of different GO biological processes, users can further select relevant sub-GO processes in a pop-up window to retrieve the corresponding chemicals. In addition, the home page provides a quick search utility, which can be used to query the database for chemicals, proteins, biological processes, or diseases. In the “Browse” page, users can easily access chemicals, proteins, biological processes, and diseases by clicking on a dropdown menu, and the complete list of matched entries can be returned (Fig. 4A). To perform data retrieval, the RCDB “Search” interface is comprised of several search options, including text search for chemicals, proteins, biological processes, and diseases (Fig. 4B). To perform a chemical search, the general name, synonyms, and CID number can be used as an input (e.g., in a search for spermidine, the following term can be used: “spermidine”). A specific protein can be queried using a protein name or gene name (e.g., a search for P07686 can be performed using “P07686” or “HCC7”). With regard to biological processes, the biological process name or GO ID can be used for searching (e.g., prostatic bud formation can be queried using “Prostatic Bud Formation” or the GO ID “0060513”). In the “disease search” section, users can search for the disease name (e.g., a search for infertility can be performed using “infertility”). In a search for the biological process and disease, all of the biological processes and diseases annotated in the database are below the search boxes for references. With clicking on the catalogue, it will automatically populate the content into the search box. And after clicking the “Go!” button, the search engine will return a result page showing comprehensive information.

In addition, the users can download all data via the “Download” page and also find a detailed tutorial on how to use the database on the “Help” page. If users have any questions, comments, or new research data and applications related to our database that have not yet been updated, they can give us feedback through the contact module.

### Data retrieval from the RCDB

**Fig. 5:**
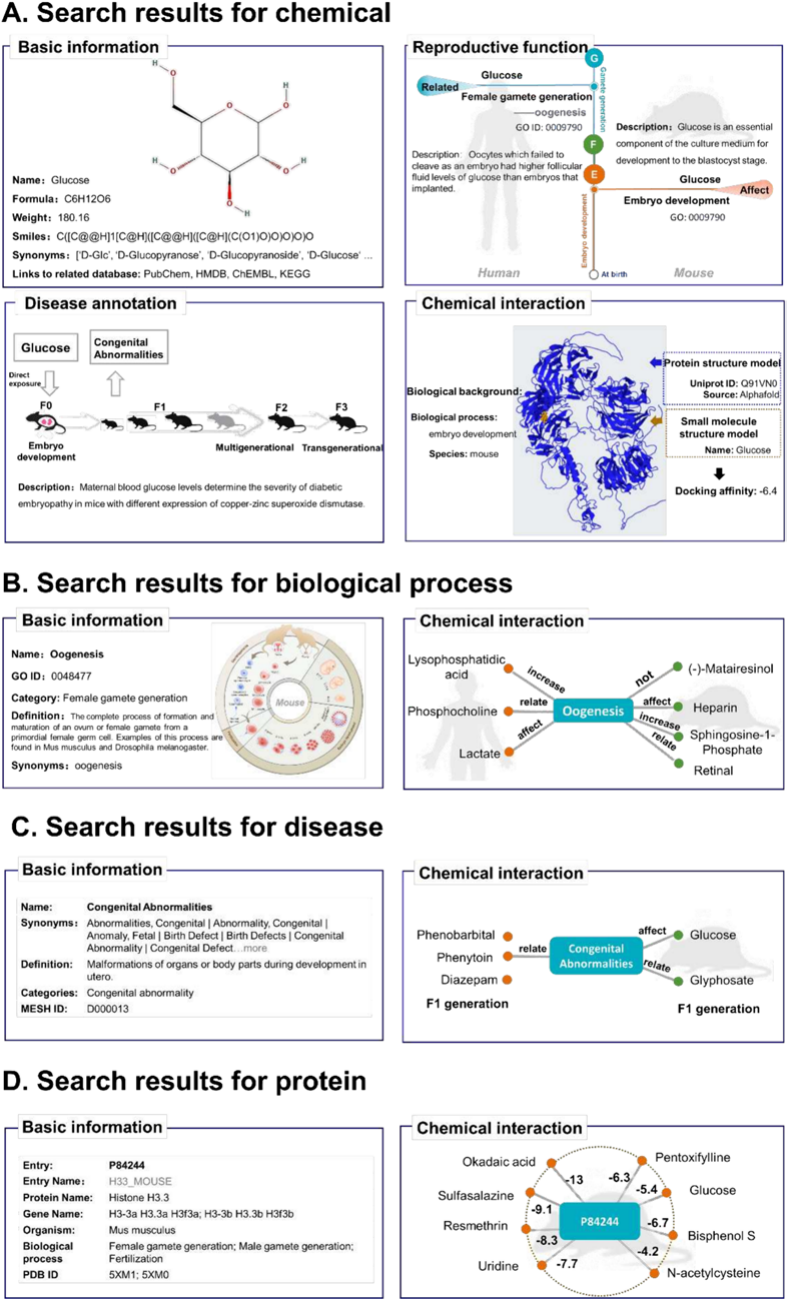
Overview of search results of the RCDB. **A** Results of a search for glucose, including basic information, reproductive function, disease annotation, and predicted protein target prediction. **B** Results of a search for the biological process of oogenesis, including basic information and a related chemical information table. **C** Results of a search for disease of congenital abnormalities, including basic information and a related chemical information table. **D** Results of a search for the protein P84244, including basic information and chemical interactions

We developed convenient web-based modules to help users quickly retrieve target information and detailed annotations from the RCDB (Fig. 5A–D). As an example of conducting targeted searches for chemicals, such as glucose, the search results include four parts, basic information including a 2D structure image, the chemical name, molecular weight, and canonical SMILES. Additionally, links to other databases, such as Human Metabolome Database (HMDB) [46] and KEGG, have been implemented on the small molecule page, enabling users to access associated target pages. The reproductive function annotation in our database encompasses chemical-induced phenotypes, action qualifiers, function periods (including crucial reproductive processes, such as female and male gamete generation, fertilization, and embryo development), and specific phenotype details with a GO ID, short description, and corresponding PubMed reference. In the related disease annotation part, the database provides related diseases of chemicals with functional biological processes, species, and generations. We also collected some endpoints, such as low birth weight, preterm birth, and perinatal complications. Low birth weight is associated with an increased risk for type 2 diabetes [47], hypertension, and cardiovascular disease. The underlying cause of low birth weight remains obscured by multiple factors, such as the potential influence of chemicals. With regard to protein target prediction, a table of predicted proteins with further detailed information is provided according to the functions of chemicals (Fig. 5A). In the search results for others, the database provides the basic information for the biological process (GO name, definition, and synonyms), disease (disease name, synonyms, categories, and Medical Subject Headings ID), and protein (Entry, Protein name, Gene name, Organism). The related chemicals are listed accordingly for all of these targets.

### Identification of chemicals within reproductive biological processes

**Fig. 6:**
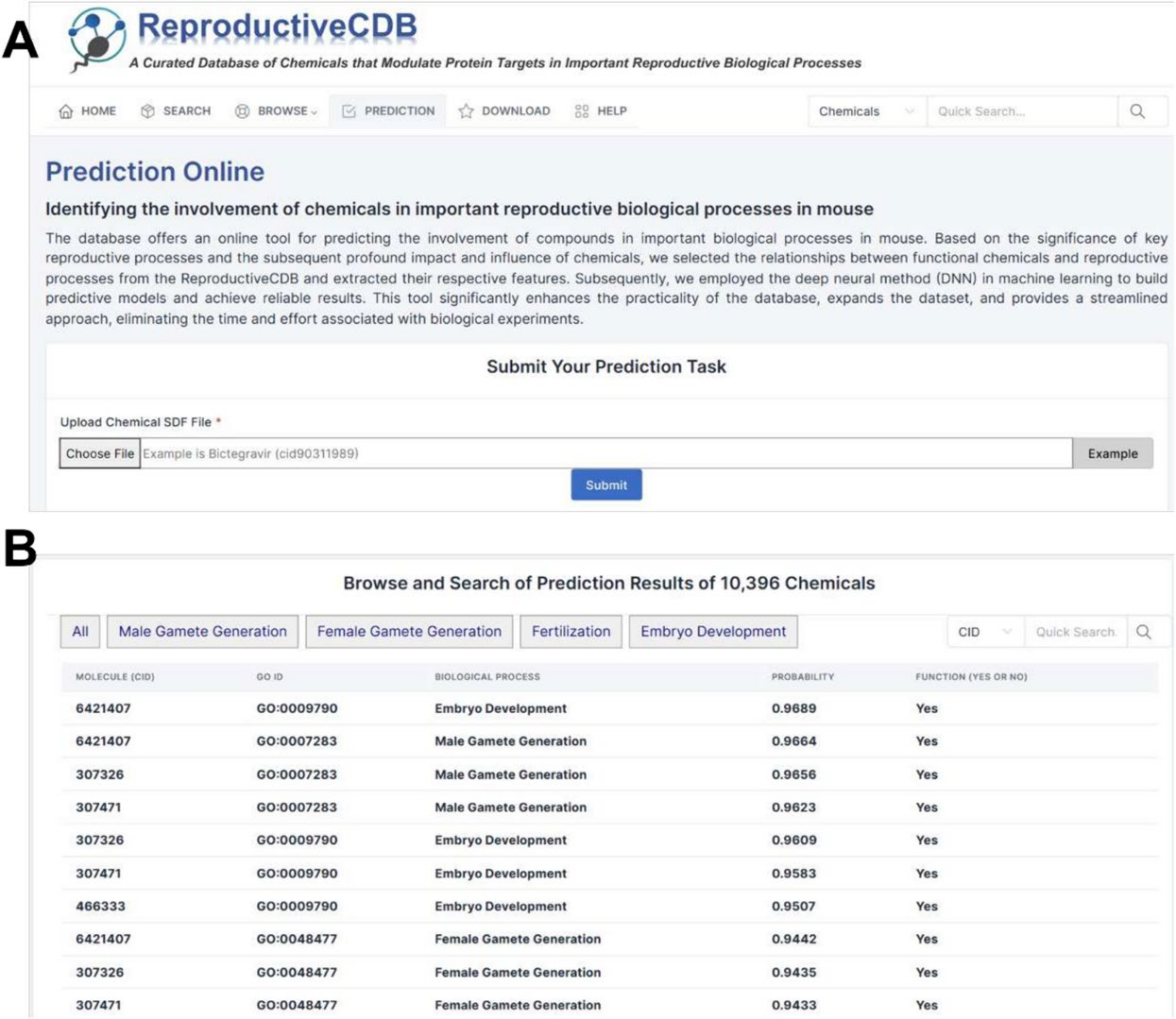
Overview of a functional module for predicting chemicals involved in reproductive biological processes. **A** Web page of the online tool. **B** List of chemicals involved in important biological processes predicted by the DNN model

In order for researchers to better identify chemicals associated with reproductive biological processes, we have predicted the involvement of 10,396 compounds that downloaded from PubChem database to find the chemicals that play roles in the important reproductive processes in the mouse. An overview of prediction result of chemicals involved in reproductive biological processes is shown in Fig. 6. This information is presented in a table format, including the compound’s CID, the biological process’s GO ID, and the correlation probability between them. A final assessment of their relevance (Yes/No) is then made. The search functionality is also supported, and users can download the table containing all predicted results of compound analysis from the “Download” page.

We compared the performance of different methods, such as deep neural network (DNN), random forest, and extreme gradient boosting. We performed tenfold cross-validation and obtained the area under the curve, Accuracy, Recall, F1-score, and Precision for each method. The average and standard deviation of the area under the curve and the area under the precision-recall curve (AUPR) from tenfold cross-validation were calculated for each method (Table 1). DNN performed the best, with the highest area under the curve of 0.7052. We built a DNN model using all chemical–reproductive biological process associations and then predicted novel associations.

**Table 1:** Performance of SVM-based PMI identification

Method	AUC	Accuracy	Precision	Recall	F1-score
XGBoost	0.6918 (0.0323)	0.6990 (0.0353)	0.5581 (0.1028)	0.1789 (0.0910)	0.2620 (0.1022)
RF	0.5796 (0.0342)	0.7138 (0.0277)	0.6363 (0.1744)	0.2221 (0.0564)	0.3233 (0.0793)
DNN	0.7052 (0.0361)	0.7208 (0.0248)	0.6711 (0.1743)	0.5741 (0.2379)	0.4663 (0.1041)

Because all known associations were used to construct the prediction model, the predicted associations require verification by published literature or other available sources. We are currently confirming the accuracy of our prediction model through two approaches. First, we select the top-ranked small molecules from the predicted results, and second, we validate them through cellular experiments.

**Fig. 7:**
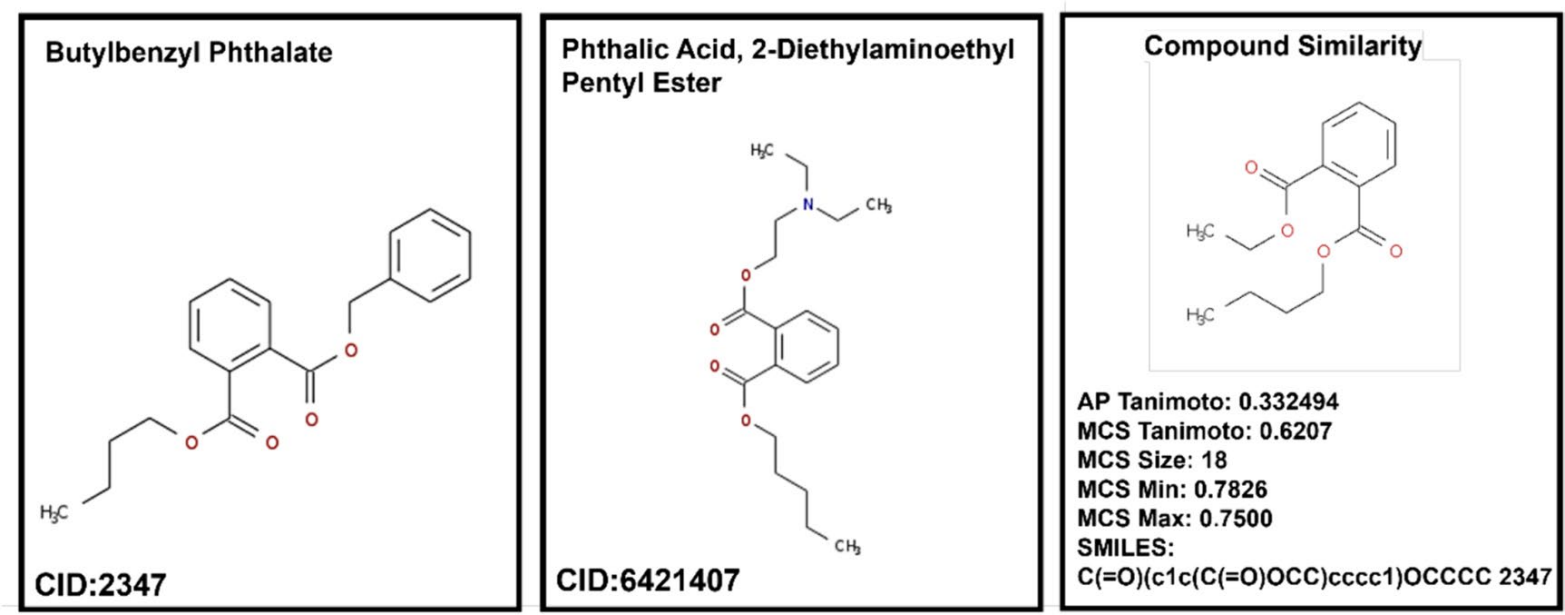
Results of chemicals structure similarity comparison

From the prediction result, “phthalic acid, 2-diethylaminoethyl pentyl ester” (CID: 6421407) is predicted to be involved in all four reproductive biological processes, with probabilities > 0.9. To investigate the principle of structurally similar molecules showing similar biological activities, we conducted a comparative analysis of structure and function between phthalic acid, 2-diethylaminoethyl pentyl ester and butyl benzyl phthalate (CID: 2347) with known reproductive activity. The parameters describe the results of a chemical structure similarity comparison (Fig. 7) analyzed by ChemMine Tools [48]. These two molecules mentioned above showed a high degree of structural similarity, and butyl benzyl phthalate showed functional involvement across all four major reproductive processes within the RCDB. This finding is in agreement with the prediction results for phthalic acid, 2-diethylaminoethyl pentyl ester in the database.

**Fig. 8:**
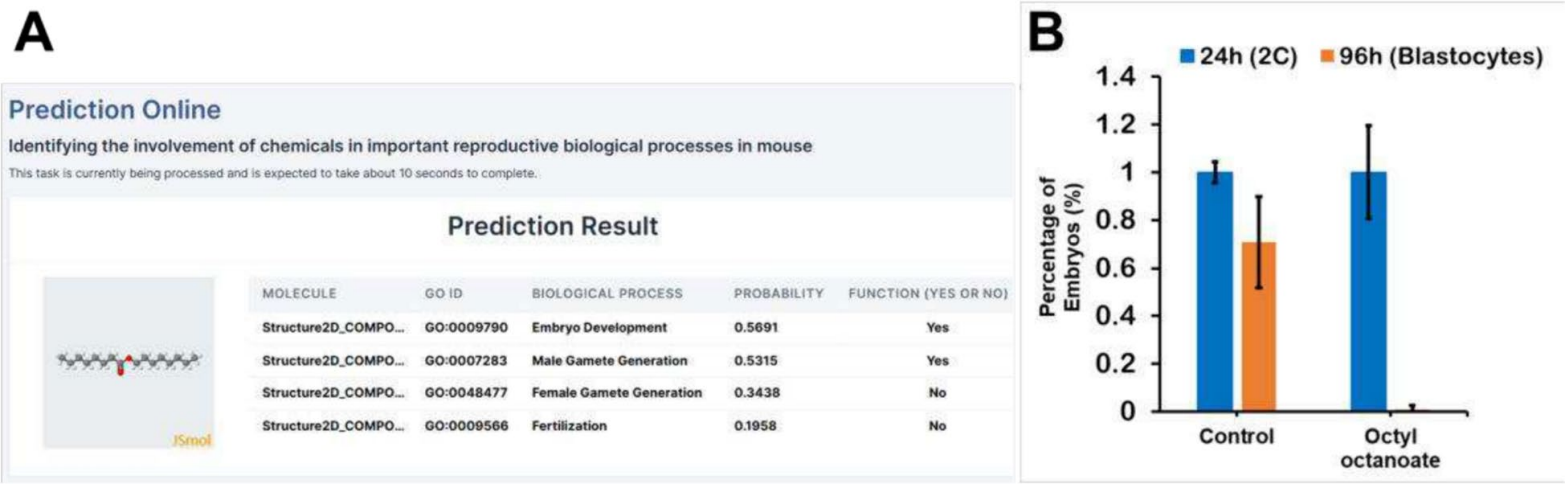
Functional roles of octyl octanoate in mouse embryos. **A** Prediction results of octyl octanoate. **B** Octyl octanoate inhibits mouse embryo development

We also identified octyl octanoate (CID: 61294) as a potential contributor to embryo development (Fig. 8A). To validate our prediction, we conducted experiments using mouse embryos. Initially, we introduced octyl octanoate into the culture medium and co-cultured it with fresh embryos collected from the pregnant mouse for 4 days. Subsequently, we assessed the developmental progress and found that octyl octanoate inhibited mouse embryo development, resulting in arrest at the two-cell to eight-cell stage (Fig. 8B). Collectively, our findings strongly support the effectiveness of our chemical function prediction model.

## Discussion

We have constructed a comprehensive, manually curated, user-friendly database (RCDB) for users to search, browse, and explore chemicals of interest. We also introduced the reverse docking method to assist researchers investigating target proteins of small molecules. This method provides a convenient reference for researchers from non-bioinformatics backgrounds and serves as a valuable supplement to current prediction databases. The RCDB is a useful resource for researchers interested in comprehending the role of chemicals in reproductive biology. This database provides a solid foundation and platform for studying research and translational applications in reproductive development.

Considering the current research status and challenges in the field of reproduction, the RCDB has the potential to be used in the following aspects. (i) The RCDB can provide a holistic view of the role of chemicals in reproductive biological processes. The users of this database can investigate basic information, relevant functions, diseases, and predicted target proteins corresponding to chemicals’ functional periods. (ii) The RCDB can provide ideas for new modulators of biological processes and potential drug candidates. Based on the predicted protein targets and biological process information, a series of new modulators (e.g., activators or inhibitors for oogenesis) might be designed and synthesized according to the known chemicals through some indirect mechanisms. (iii) The RCDB provides detailed information about related diseases. In this database, we have taken a novel approach to gathering related disease information and other endpoints, with a particular emphasis on transgenerational or multigenerational effects, which has not been reported in other databases. This information facilitates the association of diseases with relevant proteins and chemicals, which enables further studies of the underlying pathological mechanisms. Overall, in creating a comprehensive database of chemicals and their biological activities, this platform provides insights that could help guide the development of new therapeutic strategies to enhance reproductive health.

Some databases focus on important reproductive biological processes or diseases, such as the database “SpermatogenesisOnline 1.0”, which is dedicated to genes related to spermatogenesis [49], or the database “IDDB” [50], which focuses on genes related to infertility. However, these databases are limited to single biological processes or diseases and do not emphasize the study of chemicals, which may limit the diversity of their applications and the joint analysis of data. Compared with databases that focus on compounds, general databases typically have larger datasets and address broader scientific questions, which may hinder the specialization of the database’s application in the field of reproduction. An example of these differences between databases is that our target data are also reflected in other databases, such as T3DB, which aims to advance understanding on how environmental exposures affect human health [30]. In contrast to this database, we place more emphasis on metabolites that are non-toxic, but play major roles, and our annotations related to reproduction are more comprehensive. Additionally, we have added features, such as target protein prediction and molecular reproductive function prediction. Therefore, the RCDB serves as a unique resource in summarizing reproductive-related chemical data.

Further extensions of the following aspects will be conducted. First, with the advancement of technology and experimental techniques, more functional or related small molecules involved in important reproductive processes will be identified. Therefore, we will continue to track new studies and frequently update the RCDB with the addition of new chemicals’ annotations. Second, chemicals of other species, such as zebrafish, *Drosophila*, and *Caenorhabditis elegans*, which are important model animals for reproduction research, will be added in a future version of the RCDB to provide more comprehensive information for the users. Moreover, comprehensive insights on livestock will be provided to recognize the pivotal role of reproductive efficiency as a paramount determinant of their substantial economic significance. Third, we will work to optimize the “biochemical interactions” part of chemicals. The protein target prediction of the database will be improved by adding a more promising and comprehensive protein target pool. With the advent of single-cell RNA sequencing technology, researchers can systematically and accurately characterize more proteins in various biological processes or cell populations. Additionally, small molecules not only interact with proteins to function, but also act with other macromolecules, such as DNA and RNA. Therefore, we will add more interaction information in the next release. Finally, with regard to the prediction tool, we will incorporate new data or data features and optimize machine learning methods. This will further optimize the prediction methods to enhance their predictive performance, and we also explore new prediction tasks, such as predicting more specific sub-GO biological processes. Additionally, we will validate more prediction results using cellular experiments and add them to the database. Furthermore, we will add other types of analysis and applications to the database to further enhance its use and expand its scope.

## Conclusions

To our understanding, RCDB is the first uniquely dedicated database for the interpretation of the roles of chemicals in reproductive biological processes and the first database with prediction of the involvement of chemicals in GO reproductive biological processes. Taken together, our unique database holds significant importance in both biological and practical perspectives. Firstly, it facilitates the exploration and elucidation of regulatory blueprints governing important reproductive processes and pinpoint causes of many health issues. Secondly, it offers novel insights that support drug development, promote the development of the field of clinical applications in reproduction and so on.

## Data Availability

The datasets generated and analysed during the current study are all available at the website: https://yu.life.sjtu.edu.cn/ChenLab/RCDB. The implementation of prediction method and the preprocessed data is available at https://github.com/skytguuu/RCDB/tree/main.

## Abbreviations

CTD: Comparative Toxicogenomics Database
T3DB: Toxin and Toxin-Target Database
pLDDT: Predicted Local Distance Difference Test
3D: Three-dimensional
CBIs: Chemical–biological process interactions
PMSG: Pregnant mare serum gonadotropin
hCG: Human chorionic gonadotropin
HMDB: Human Metabolome Database
DNN: Deep neural network
AUPR: Area under the precision-recall curve
